# Bibliometric analysis of immunotherapy for esophageal cancer: 2004-2024

**DOI:** 10.3389/fonc.2025.1565132

**Published:** 2025-09-02

**Authors:** Yunqi Hua, Yubo Liu, Yanhong Liu, Xiaoling Tian, Xinyi Zhang, Jiacong You, Fangrui Yin

**Affiliations:** ^1^ Department of oncology, Baotou Cancer Hospital, Baotou, Inner Mongolia, China; ^2^ Baotou Medical College, Inner Mongolia University of Science and Technology, Baotou, Inner Mongolia, China; ^3^ Editorial Office of Medical Journal of Peking Union Medical College Hospital, Peking Union Medical College Hospital, Chinese Academy of Medical Sciences & Peking Union Medical College Hospital, Beijing, China; ^4^ Department of Rheumatology, The First Affiliated Hospital of Baotou Medical College, Baotou, Inner Mongolia, China

**Keywords:** esophageal cancer, immunotherapy, bibliometrics, visualization, PD-1

## Abstract

Esophageal cancer (EC) remains one of the most lethal malignancies worldwide, with particularly high incidence and mortality rates in developing countries. In recent years, immunotherapy has emerged as a promising treatment strategy. This study employed bibliometric visualization tools, including CiteSpace and VOSviewer, to analyze research pertaining to immunotherapy in esophageal cancer. Based on 780 English-language publications indexed in the Web of Science database from 2004 to 2024, we quantitatively examined the research landscape, evolutionary trends, and knowledge structure of this field. The results indicated a steady increase in annual publications, with China being the leading contributor and Zhengzhou University as the most productive institution. *Frontiers in Oncology* and *Journal of Clinical Oncology* were the most prolific and most co-cited journals, respectively, while Kato Ken and Kojima Takashi were identified as the most influential scholars in the field. The research focus has shifted from traditional radiotherapy and chemotherapy to immunotherapy-related areas, such as the tumor immune microenvironment, immune checkpoint inhibitors, PD-L1 expression, and microsatellite instability. Current research emphasizes combining immunotherapy with other modalities, including radiotherapy, chemotherapy, and targeted therapy, pointing toward future multimodal combination strategies. Additionally, efforts are directed at identifying novel biomarkers to enhance treatment efficacy, reduce toxicity, and improve patient survival.

## 1 Introduction

Esophageal cancer is a malignant tumor characterized by high global incidence and mortality rates. Its primary pathological types include esophageal squamous cell carcinoma and esophageal adenocarcinoma (EAC). Although recent years have witnessed certain progress in traditional treatment modalities such as surgery, radiotherapy, and chemotherapy, the overall prognosis for advanced or recurrent esophageal cancer remains poor, with a five-year survival rate below 20%. Consequently, exploring novel therapeutic approaches to improve patient outcomes has become a major focus of medical research. In recent years, alongside in-depth investigations into the tumor immune microenvironment (TIME) and immune escape mechanisms, immunotherapy has significantly improved progression-free survival (PFS) in patients with various malignancies. Compared to traditional chemotherapy, immunotherapy is associated with a marked reduction in grade 3 or 4 adverse events (1, 2). Treatment strategies represented by ICIs activate the body’s anti-tumor immune response by targeting key pathways such as PD-1(Programmed Cell Death Protein 1), PD-L1, and CTLA-4(Cytotoxic T-Lymphocyte Associated Protein 4) (3, 4). This approach achieved its first and most significant success in non-small cell lung cancer (NSCLC) (5, 6) and has also yielded remarkable results in other solid tumors, including melanoma and gastric cancer (7, 8), offering a promising new direction for the treatment of esophageal cancer. For esophageal cancer specifically, its distinct immune microenvironment features, such as high rates of PD-L1 and PD-L2(Programmed Death-Ligand 2) expression, offer potential targets for immunotherapy (9). Preliminary clinical trial results indicate that ICIs(immune checkpoint inhibitors) demonstrate favorable safety and promising efficacy in the treatment of esophageal cancer (10). For instance, the KEYNOTE-181 trial by Kojima et al. first demonstrated that pembrolizumab was superior to chemotherapy in the second-line treatment of advanced esophageal cancer with PD-L1-positive tumors (11). Furthermore, the CheckMate 577 trial established that nivolumab as adjuvant therapy significantly prolonged disease-free survival (DFS) in patients following curative resection (12), representing a breakthrough in clinical translation. However, variations in immunotherapy response based on different pathological subtypes, tumor stages, and biomarkers, as well as the occurrence of primary or acquired resistance in some patients, remain critical unresolved issues. These clinical challenges, including differential treatment responses, the complexity of resistance mechanisms, and the heterogeneity and inconsistency in the predictive value of biomarkers, continue to demand urgent solutions (13). Bibliometrics, a tool combining mathematical and statistical methods to qualitatively and quantitatively evaluate academic literature within a specific field, enables the analysis of its development and trends from a global perspective (14). Currently, as an emerging methodology, it has been widely applied across numerous medical fields, such as targeted therapy for cholangiocarcinoma (15). Furthermore, Chen et al. developed an AI-powered bibliometric model (SciBERT) in 2023 capable of automatically identifying interdisciplinary research hotspots (16). Taking immunotherapy for gastrointestinal tumors, as discussed in this work, as an example: bibliometric analyses already exist for immunotherapy in gastric cancer and hepatocellular carcinoma (16, 17). Notably, a comprehensive bibliometric study analyzing the global developmental trajectory of immunotherapy specifically for esophageal cancer (encompassing both ESCC and EAC) is currently lacking. This study pioneers the application of multidimensional bibliometric analytical tools to the field of esophageal cancer immunotherapy, thereby addressing a critical research gap specifically in this domain. To address this gap, we conducted a bibliometric analysis of the global research landscape in esophageal cancer immunotherapy from 2004 to 2024 using multiple analytical tools (VOSviewer, CiteSpace, Ch articulator). This analysis aimed to: 1) Quantify national/institutional collaboration networks, examining the evolution of publication growth, national contributions, and collaborative patterns within the field; 2) Identify evolving research hotspots, particularly how the focus has shifted from traditional therapies (chemoradiotherapy) towards immunotherapy and combination therapies; 3) Predict future research directions, such as the exploration of biomarkers and overcoming resistance mechanisms. Our findings reveal that the PD-1/PD-L1 axis is central to immunotherapy, with PD-L1 expression associated with poor prognosis in esophageal cancer (18). The binding of PD-1 to PD-L1 inhibits T-cell generation, thereby promoting tumor escape (19). Currently, combination therapy and the exploration of novel biomarkers have become dominant trends in esophageal cancer immunotherapy. Trials such as Sun et al.’s KEYNOTE-590 (20) and Doki et al.’s CheckMate 648 have demonstrated the efficacy and reliability of combination regimens, establishing them as the future direction for esophageal cancer treatment (21). Furthermore, the team led by Xu Ruihua demonstrated the predictive value of tumor mutational burden, microsatellite instability, and EGIC(Esophageal cancer Genome-based Immuno-oncology Classification) typing for immunotherapy response (22, 23).

## 2 Methods

### 2.1 Data retrieval

Web of Science (WOS) is a high-quality digital publication resource database that has been widely accepted by researchers for bibliometric analysis. On December 2, 2024, we retrieved all publications related to esophageal cancer immunotherapy from January 1, 2004, to December 2, 2024, in the WOS core database. The screening date for articles was December 25, 2024. To ensure data timeliness, all data used the information retrieved on that day to avoid causing bias. The search formula was as follows:

TS=[("Esophageal Neoplasm" OR "Neoplasm, Esophageal" OR "Esophagus Neoplasm" OR "Esophagus Neoplasms" OR "Neoplasm, Esophagus" OR "Neoplasms, Esophagus" OR "Neoplasms, Esophageal" OR "Cancer of Esophagus" OR "Esophageal Cancer" OR "Cancer, Esophageal" OR "Cancers, Esophageal" OR "Esophageal Cancers" OR "Cancer of the Esophagus" OR "Esophagus Cancer" OR "Cancer, Esophagus" OR "Cancers, Esophagus" OR "Esophagus Cancers") AND ("Immunotherapy" OR "Immunotherapies")]

#### 2.1.1 Inclusion criteria

1. The subject must be esophageal cancer immunotherapy, with full-text availability;
2. Written in English;
3. Publication period from 2004 to 2024;
4. Article types: original articles and reviews.

#### 2.1.2 Exclusion criteria

1. Studies with non-relevant research topics (i.e., not focused on esophageal cancer immunotherapy) or unavailable full-text were excluded.
2. Publications in non-English languages were excluded.
3. Publications outside the 2004–2024 timeframe were excluded.
4. Publications of any type other than original articles and reviews were excluded.

### 2.2 Software analysis

Bibliometric methods were employed to collect frontier knowledge and trends in the research field. In this study, three visualization tools were used: VOSviewer, CiteSpace, and an online platform, to predict trends in esophageal cancer immunotherapy.

VOSviewer is a visualization tool for scientific bibliometric analysis, widely used in literature analysis, scientometrics, and network analysis. It was developed by the Science Metrix & Knowledge Visualization Group at Leiden University and is primarily used to generate various types of charts and network maps based on bibliometric data, helping researchers identify relationships and trends between publications. Its strengths lie in exceptional visualization capabilities and highly efficient clustering algorithms, demonstrating particular proficiency in unveiling static knowledge structures and collaborative networks within large-scale bibliometric datasets. In this study, we used VOSviewer to analyze institutions, journals, authors, keywords, and highly cited papers. VOSviewer version 1.6.20 was utilized for the analysis.

CiteSpace is a widely utilized tool in scientometrics and visual analytics, particularly adept at conducting research domain trend analysis, knowledge mapping, and literature co-citation analysis. Its distinction lies in robust time-series analytical capabilities, excelling at unveiling the dynamic evolutionary trajectories, developmental trends, and emergent frontiers within research fields, thematic areas, conceptual frameworks, or technological domains. Developed by Professor Chaomei Chen, CiteSpace is mainly used to identify research hotspots, development trends, key publications, and important research frontiers in a discipline. It supports in-depth analysis of research data from multiple perspectives, helping researchers understand the structure and evolution of academic fields. In this study, we used CiteSpace for dual-map overlay analysis of journals, keyword timeline views, keyword bursts, co-citation analysis, and co-citation burst analysis. Utilizing CiteSpace software (version 6.3.R1) for bibliometric network visualization, we configured the Pathfinder network pruning parameter at K = 25 with a time slicing span from 2005 to 2024.

In addition, we also utilized the online platform Charticulator, an interactive data visualization tool developed by Microsoft Research. Charticulator allows users to create custom interactive data visualizations through a drag-and-drop interface without the need for programming knowledge. It is especially suitable for users who wish to have precise control over the style and layout of data visualizations. In this study, we used Charticulator to create a network map of relationships between countries.

## 3 Result

**Figure 1:**
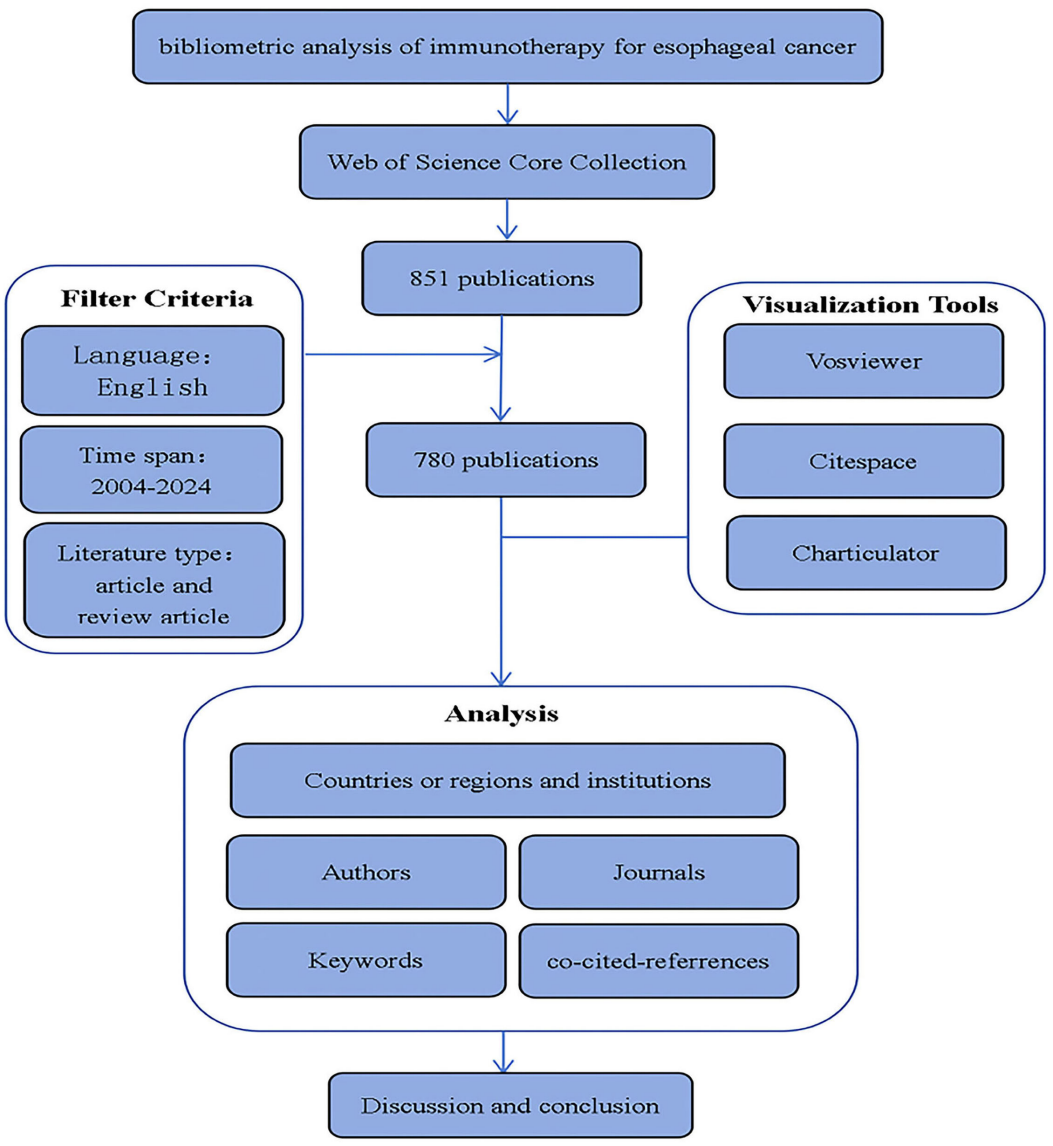
flowchart of the article.

Trends in annual growth of publications and citations on esophageal cancer Immunotherapy. According to the inclusion criteria (**Figure 1**, Flowchart of the article), from January 1, 2004, to December 2, 2024, a total of 780 articles on esophageal cancer immunotherapy were published in Web of Science (WOS). Of these, 476 (61%) were original research articles and 304 (39%) were reviews. The published literature spans 52 countries or regions, with the majority of publications originating from China, the United States, and Japan. The most frequently published authors were Ken Kato and Takashi Kojima. The article with the highest citation count was “Nivolumab versus chemotherapy in patients with advanced oesophageal squamous cell carcinoma refractory or intolerant to previous chemotherapy (ATTRACTION-3): a multicentre, randomized, open-label, phase 3 trial”. The journal with the most published articles was Frontiers in Oncology.

**Figure 2:**
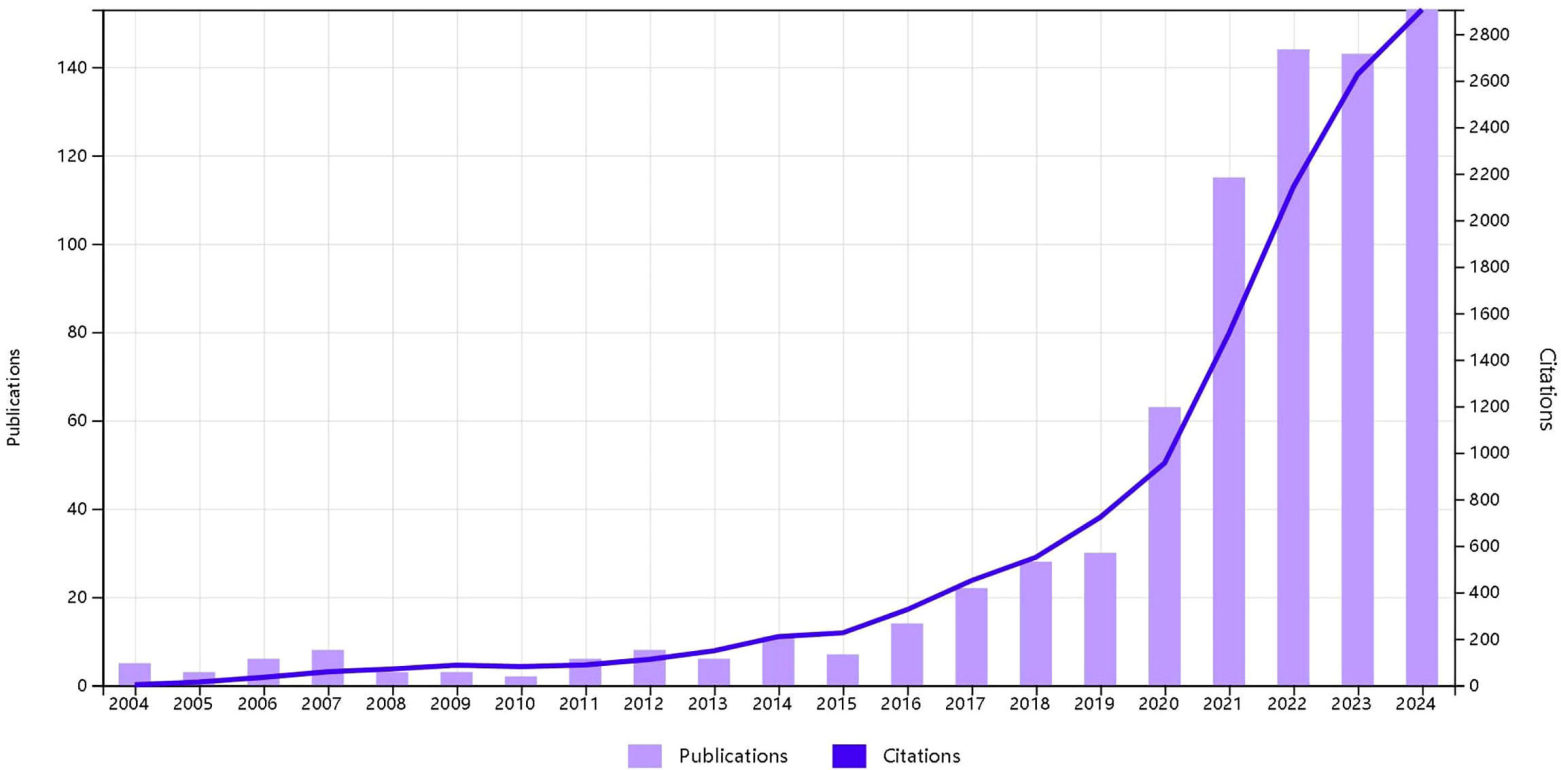
Annual trends of articles published in immunotherapy for EC from 2004 to 2024.

Before 2019, the research trend line and citation growth remained relatively flat. However, after 2019, both the annual number of publications and citations increased rapidly, particularly in 2024, which saw the highest publication and citation counts. The number of publications and citations related to esophageal cancer immunotherapy from 2004 to 2024 is shown in (**Figure 2**, Temporal trend of publications).

### 3.1 Analysis of countries/regions and institutions

**Figure 3:**
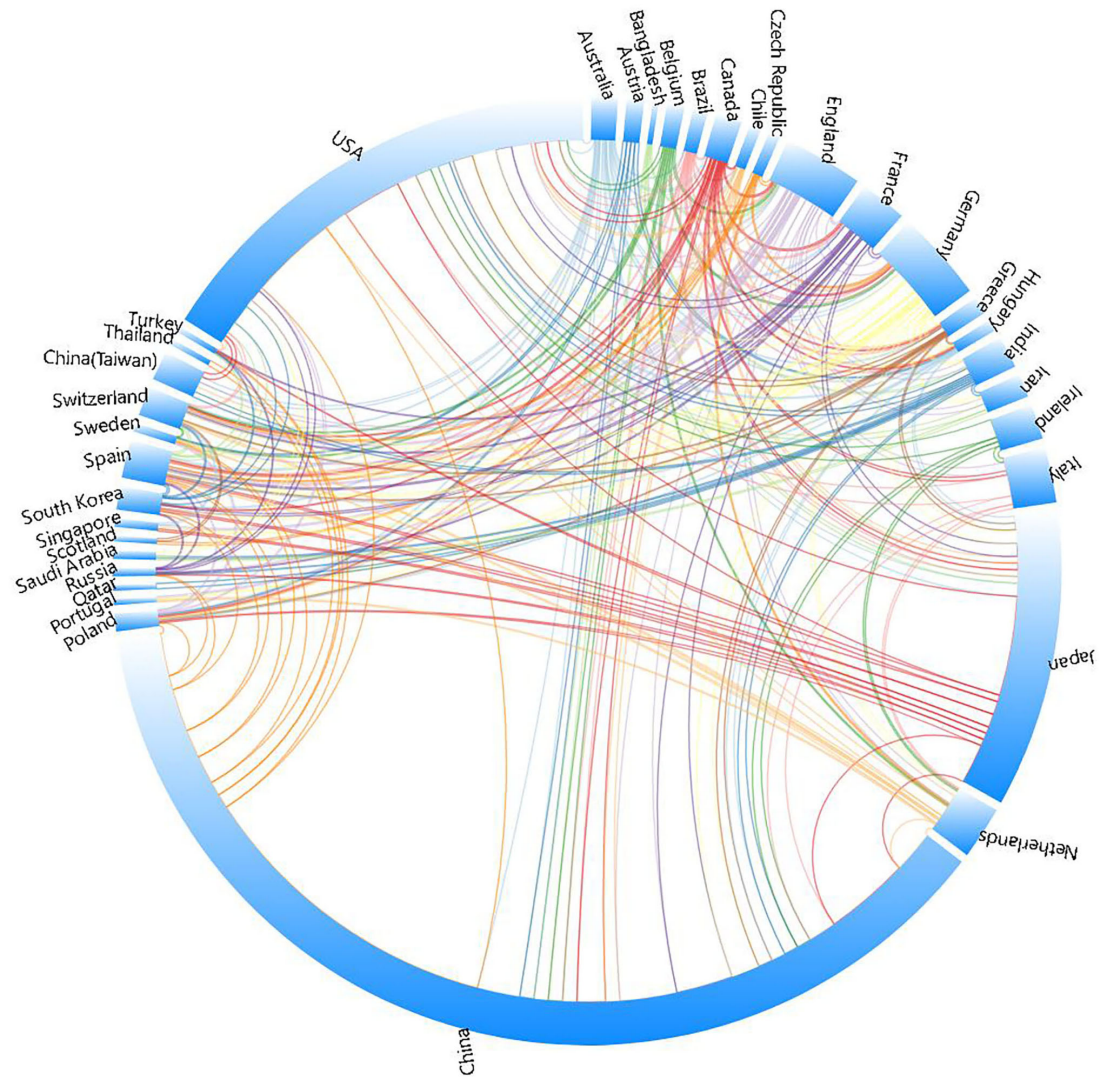
A visualization of countries related to immunotherapy for EC.

China emerged as the most prolific publisher, contributing 370 articles (47.4% of the total), followed by the United States with 159 articles (20.4%), and Japan with 101 articles (12.9%). China’s output alone constitutes nearly half of all publications, demonstrating an absolute scale advantage. Collectively, these three nations account for 80.7% of total publications. **Figure 3** illustrates that from 2004 to 2024, China, the US, and Japan—the top three publishing countries—have consistently driven research in esophageal cancer immunotherapy, with colored lines indicating collaborative linkages between nations(**Figure 3**, Country collaboration network).

Among the top 10 publishing countries/regions (**Table 1**), China also leads in total citations (5,358), followed by Japan (3,740) and the United States (3,241). However, China’s citation per article ratio (14.48) ranks only 22nd globally. Notably, Singapore achieved the highest citation per article ratio worldwide (67.33), trailed by Turkey (65.00) and Russia (62.67), highlighting significant disparities in research efficiency.

**Table 1:** The top 10 most published countries/regions related to immunotherapy for EC from 2004 to 2024.

Rank	Country/region	Article counts	Citation	Citation per articles
1	China	370	5358	14.48
2	USA	159	3241	20.38
3	Japan	101	3740	37.03
4	Germany	31	611	19.71
5	England	27	711	26.33
6	Italy	19	254	13.37
7	Netherlands	18	364	20.22
8	France	16	370	23.13
9	Spain	13	543	41.77
10	Ireland	12	136	13.58

**Figure 4:**
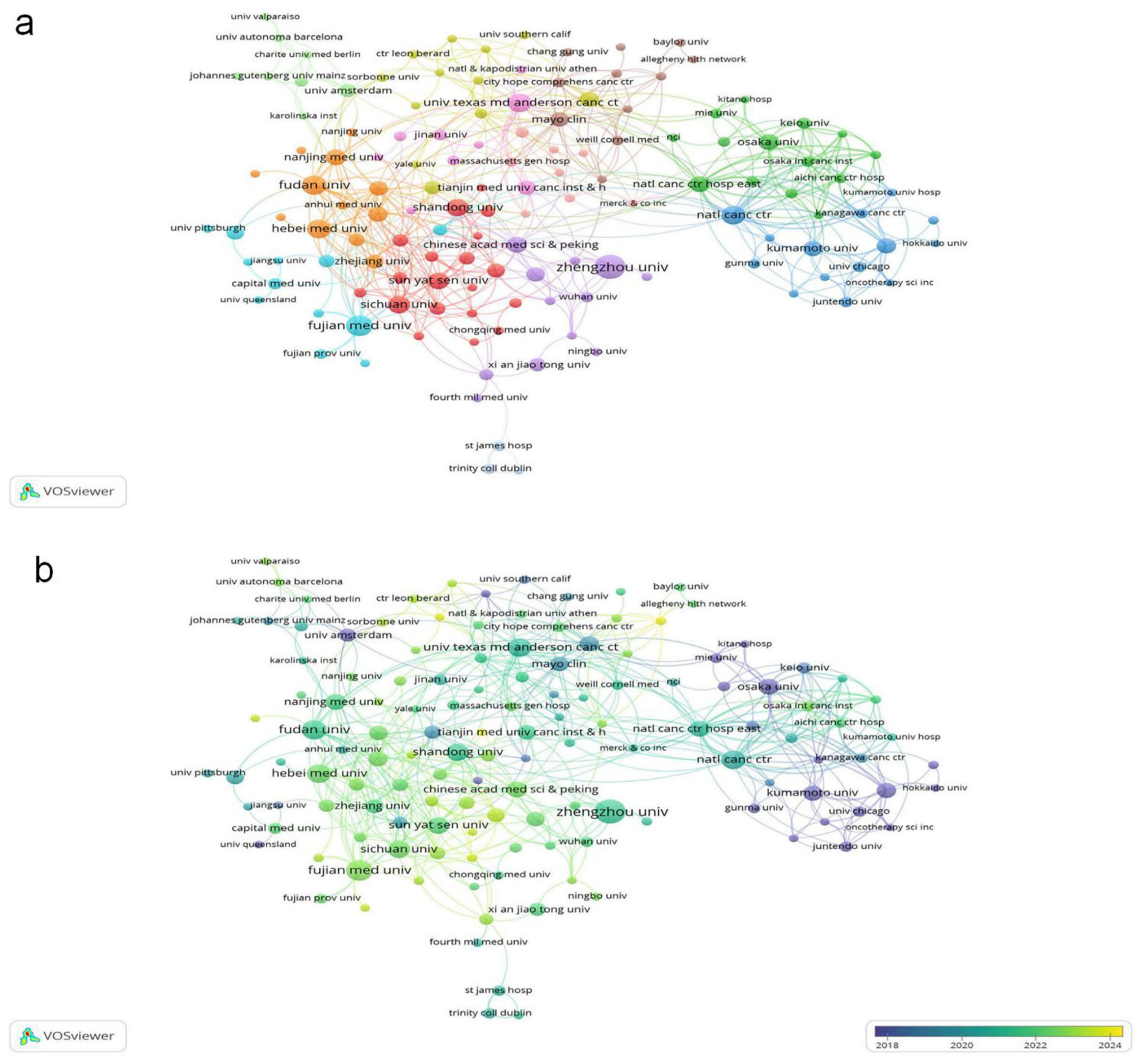
**(a)** Cooperative network of publications between institutions. Nodes represent institutions, and lines indicate connections. The larger the node, the higher the publication volume. **(b)** From blue to yellow, the closer the color is to yellow, the later the primary publication time.

Institutional analysis reveals Chinese dominance: 8 of the top 10 publishing institutions are from China, underscoring the nation’s concentrated focus on this field. Zhengzhou University leads with 30 publications (**Table 2**), followed by Fujian Medical University and Fudan University (**Figure 4a**, Institutional collaboration network diagram; **Figure 4b**, Institutional temporal trend diagram). Among all institutions, Juntendo University garnered the highest total citations (898), while Shandong Cancer Hospital & Institute achieved the top citation per article ratio (194.33).

**Table 2:** The top 10 most published institutions related to immunotherapy for EC from 2004 to 2024.

Rank	Institution	Article counts	Citation	Citation per articles
1	Zhengzhou univ	30	478	15.93
2	Fujian med univ	23	119	5.17
3	Fudan univ	20	268	3.40
4	univ Texas md Anderson canc ctr	19	360	18.95
5	Hebei med univ	18	99	5.50
6	natl canc ctr	18	501	27.83
7	Shandong univ	16	711	44.44
8	Sichuan univ	16	233	14.56
9	Sun Yyat Sen univ	15	224	14.93
10	Chinese acad med sci & Peking union med coll	14	202	14.43

### 3.2 Journal analysis

**Figure 5:**
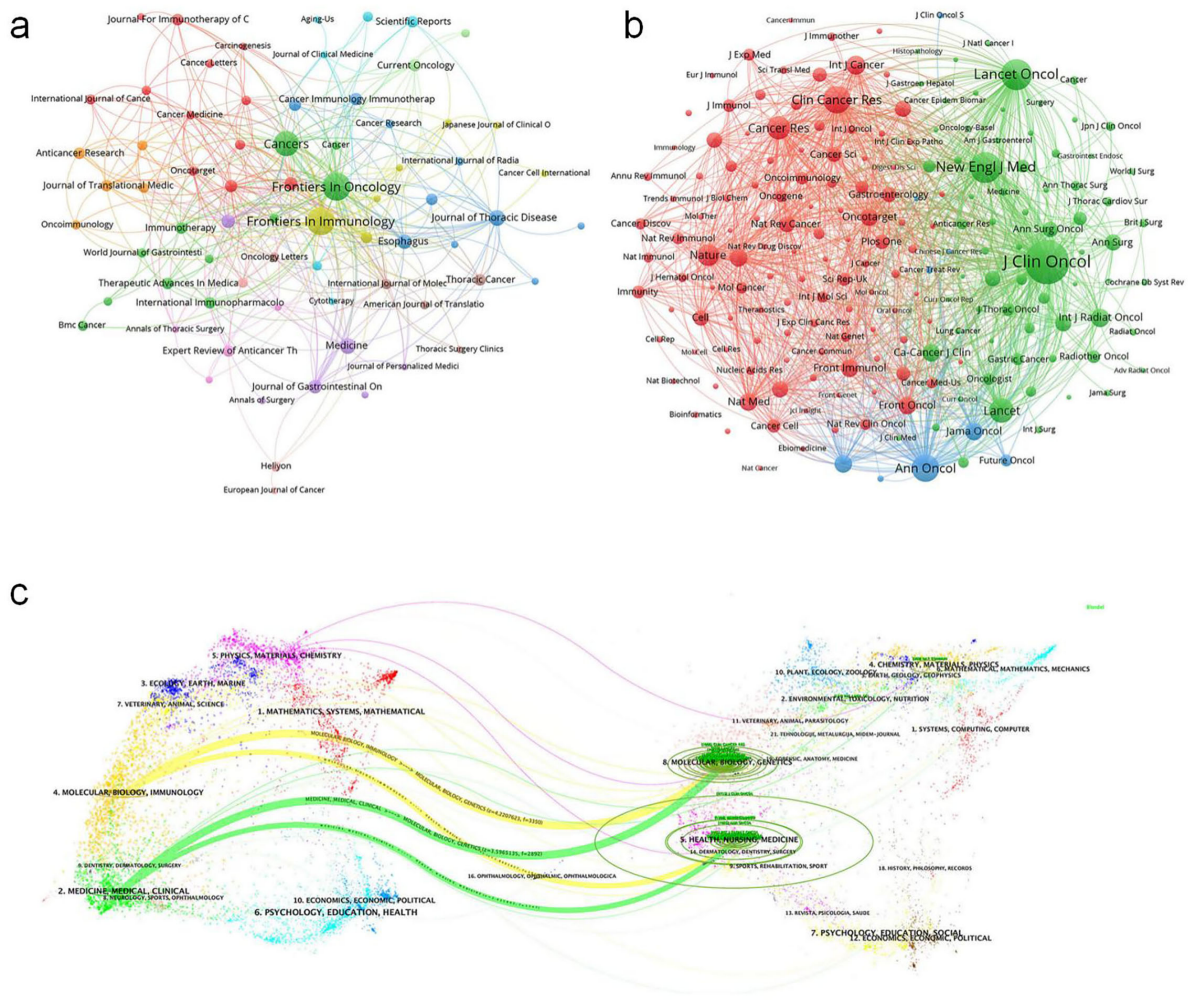
**(a)** Journals visualization network map, where nodes represent journals, lines represent connections, and the larger the node, the higher the publication volume. **(b)** Journals visualization network map, where nodes represent journals, lines represent connections, and the larger the node, the higher the citation volume. **(c)** Biplot of published and cited literature journals, with published journals on the left and cited journals on the right.

Frontiers in Oncology published the highest number of articles (42, 5.38% of total publications) in esophageal cancer immunotherapy research, followed by Frontiers in Immunology (41, 5.26%), Cancers (34, 4.36%), Journal of Thoracic Disease (14, 1.79%), and Medicine (13, 1.67%) (**Figure 5a**, High-Volume Journal Network Diagram). Among the top 10 publishing journals, Clinical Cancer Research had the highest impact factor (9.0), with Frontiers in Immunology (5.7), Cancers, and Cancer Science (both 4.5) following. Geographically, 30% of these top 10 journals originated from China, 30% from Switzerland, 20% from the United States, 10% from the United Kingdom, and 10% from Germany. Regarding Journal Citation Reports (JCR) quartiles, 30% were classified as Q1, 40% as Q2, and 30% as Q3. Clinical Cancer Research received the highest total citations (1,453), trailed by Frontiers in Oncology (541). It also achieved the highest citation-per-article ratio (181.6), followed by Cancer Science (46.0) (**Figure 5b**, Highly Cited Journals Network Diagram). Notably, despite having the lowest publication volume among analyzed journals, Clinical Cancer Research led in both citation metrics, reflecting the exceptional quality of its output (**Table 3**).

**Table 3:** The top 10 most published journals related to immunotherapy for EC from 2004 to 2024.

Rank	Journal	Article counts	Citation	Citation per articles	JCR partition	IF
1	Frontiers In Oncology	42	541	12.88	Q2	3.50
2	Frontiers In Immunology	41	492	12.00	Q1	5.70
3	Cancers	34	247	7.26	Q1	4.50
4	Journal of Thoracic Disease	14	104	7.43	Q2	2.10
5	Medicine	13	37	2.85	Q2	1.30
6	Esophagus	11	153	13.91	Q3	2.20
7	Cancer Science	10	117	11.70	Q3	2.00
8	Journal of Gastrointestinal Oncology	10	460	46.00	Q2	4.50
9	Annals of Translational Medicine	9	81	9.00	Q3	3.93
10	Clinical Cancer Research	8	1453	181.60	Q1	9.00

For journal co-citation analysis, Journal of Clinical Oncology ranked first (2,715 co-citations), followed by New England Journal of Medicine (1,337) and Lancet Oncology (1,303) (**Table 4**). All top 10 co-cited journals belonged to Q1, with 50% based in the US and 50% in the UK. The Lancet possessed the highest impact factor (98.4) in this group. A dual-map overlay illustrates the thematic distribution of journals (**Figure 5c**, Biplot of published and cited literature journals).

**Table 4:** The top 10 most cited journals related to immunotherapy for EC from 2004 to 2024.

Rank	Journal	Citation	JCR partition	IF
1	J Clin Oncol	2715	Q1	42.10
2	New Engl J Med	1337	Q1	21.69
3	Lancet Oncol	1303	Q1	41.60
4	Clin Cancer Res	1066	Q1	12.53
5	Ann Oncol	1025	Q1	18.81
6	Lancet	858	Q1	98.40
7	Cancer Res	745	Q1	12.10
8	Nature	660	Q1	19.40
9	Int J Cancer	505	Q1	5.70
10	Int J Radiat Oncol	465	Q1	6.40

### 3.3 Author analysis

**Figure 6:**
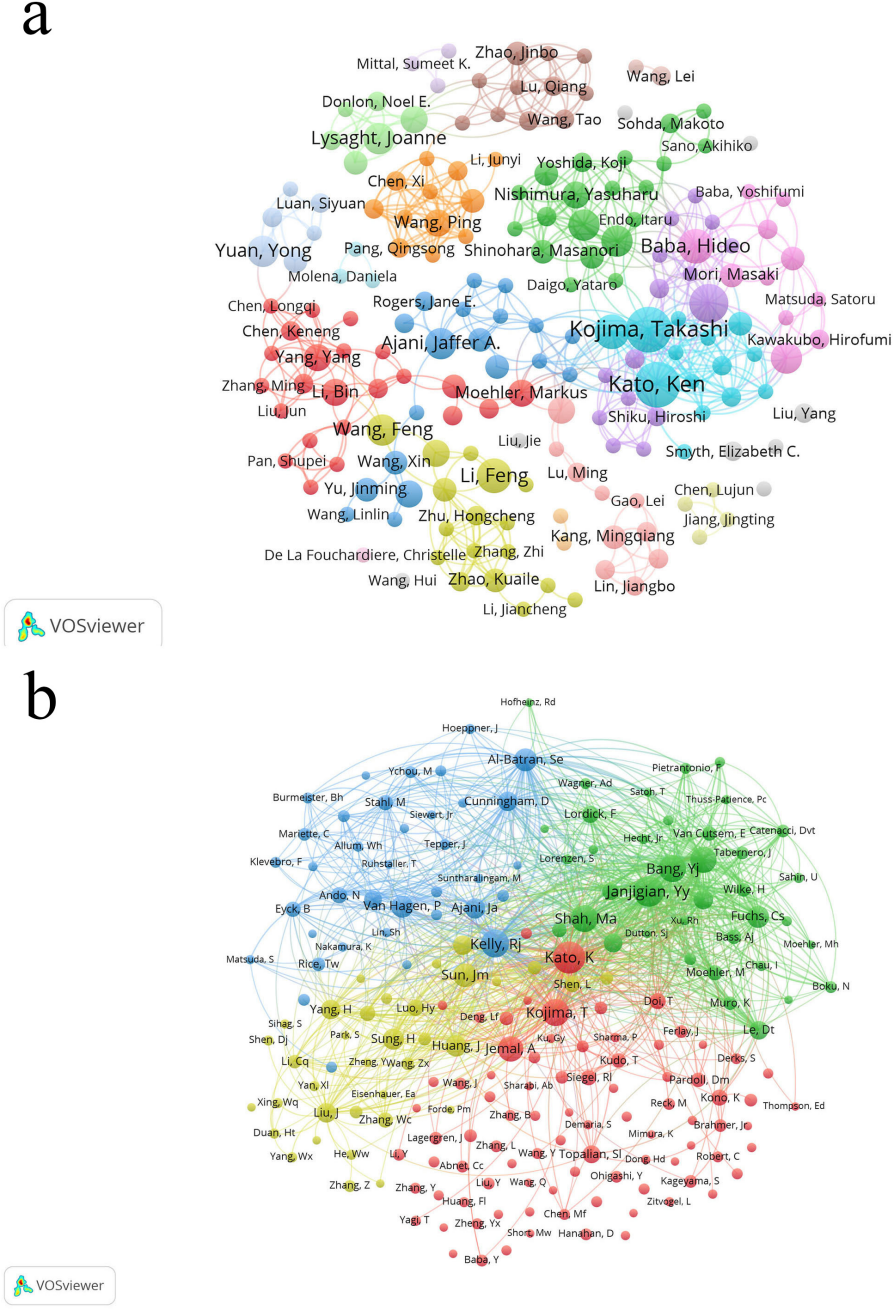
**(a)** The larger the node, the higher the publication volume. **(b)** The larger the node, the higher the total citation frequency.

From 2004 to 2024, approximately 4500 researchers were involved in studies related to esophageal cancer immunotherapy. Among them, the authors with the most publications are Kato, Ken, and Kojima, Takashi, with 11 papers each, followed by Doki, Yuichiro (9 papers), and Baba, Hideo, Doi, Toshihiko, and Li, Feng (8 papers) (**Table 5**). As shown in **Figure 6a** (**Figure 6a**, High-Volume authors collaboration network diagram), the larger the node, the higher the number of publications. The authors with the highest citation counts are Yu, Jinming (677 citations), Wang, Xin (624), and Nakamura, Yusuke (567). The authors with the highest citation/publication ratios are also Yu, Jinming (135.4), followed by Wang, Xin (124.8) and Daigo, Yataro (95). The most cited author overall is Kato, Ken (278 citations), followed by Janjigian, Yy (244) and Kelly, Rj (205) (**Figure 6b**, Authors’ network diagram with high average citations per publication). It is noteworthy that Kato, Ken not only has the highest number of publications but also the highest citation count, indicating that Kato, Ken’s research output is not only quantitatively significant but also widely recognized for its quality and academic influence.

**Table 5:** The top 10 most published authors related to immunotherapy for EC from 2004 to 2024.

Rank	Author	Article counts	Citation	Citation per articles
1	Kato, Ken	11	291	26.45
2	Kojima, Takashi	11	471	42.82
3	Doki, Yuichiro	9	173	19.22
4	Baba, Hideo	8	340	42.50
5	Doi, Toshihiko	8	499	62.38
6	Li, Feng	8	79	9.88
7	Ajani, Jaffer A.	7	567	81.00
8	Kitagawa, Yuko	7	109	15.57
9	Lysaght, Joanne	7	147	21.00
10	Nakamura, Yusuke	7	352	50.29

### 3.4 Keyword analysis, keyword timeline view and keyword burst analysis

**Figure 7:**
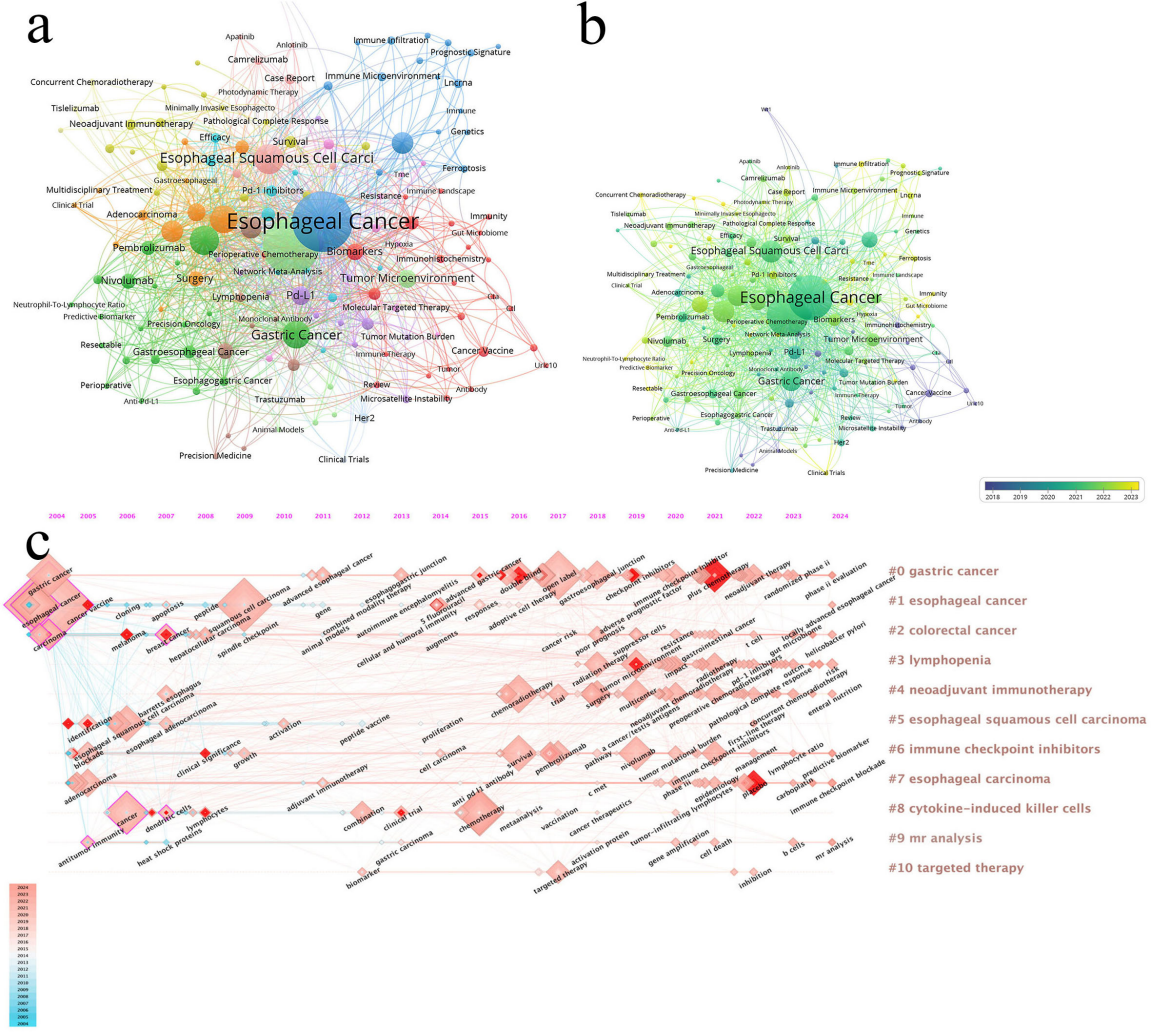
**(a)** The larger the node, the higher the frequency of occurrence. **(b)** From blue to yellow, the closer it is to yellow, the later the occurrence time. **(c)** Visualization map of timeline viewer related to immunotherapy for EC, produced by CiteSpace.

After deduplication of keywords using Vosviewer, **Table 6** reveals that the most frequently occurring keywords are “esophageal cancer,” “immunotherapy,” “Esophageal Squamous Cell Carcinoma,” “Immune Checkpoint Inhibitors,” and “Chemotherapy.” Excluding disease-related keywords, the most common treatment-related terms include “immunotherapy,” “immune checkpoint inhibitors,” “chemotherapy,” “Neoadjuvant Therapy,” and “Radiotherapy” (**Figure 7a**, Keyword network diagram). The importance of keywords is evident, as they reflect the evolution of cutting-edge knowledge and technology over time (**Figure 7b**, Keyword trend visualization diagram), which is crucial for guiding future research directions in the field (16). Our analysis of the most frequently occurring keywords reveals three primary themes: 1. esophageal cancer and its specific subtypes, 2. Immunotherapy, and 3. Diverse therapeutic approaches for esophageal cancer. This clearly indicates that the treatment of esophageal cancer must be based on precision-based and personalized strategies according to different subtypes, while the emergence of immunotherapy has catalyzed the momentum toward combination therapies.

**Table 6:** The top 20 most frequent keywords related to immunotherapy for EC form 2004 to 2024.

Rank	Keyword	Occurrences
1	Esophageal Cancer	355
2	Immunotherapy	306
3	Esophageal Squamous Cell Carcinoma	87
4	Immune Checkpoint Inhibitors	83
5	Chemotherapy	77
6	Gastric Cancer	77
7	Neoadjuvant Therapy	48
8	Prognosis	46
9	Radiotherapy	43
10	Pd-L1	36
11	Chemoradiotherapy	33
12	Tumor Microenvironment	31
13	Biomarkers	27
14	Pd-1	25
15	Targeted Therapy	25
16	Nivolumab	23
17	Surgery	22
18	Pembrolizumab	20
19	Meta-Analysis	19
20	Squamous Cell Carcinoma	19

The timeline view is used to display how events or data points change over time, providing a clear representation of temporal variations (**Figure 7c**, Timeline visualization diagram). It helps researchers identify trends and periodicity, allowing for a comparative analysis of data across different time periods. From the figure, we observe that “#1 esophageal cancer” appeared earliest. “#4 neoadjuvant immunotherapy” and “#6 immune checkpoint inhibitors” began to gain popularity around 2016. Recent research hotspots include topics such as PD-1 inhibitors (e.g., nivolumab), tumor immune microenvironment, and immune checkpoint inhibitors.

**Figure 8:**
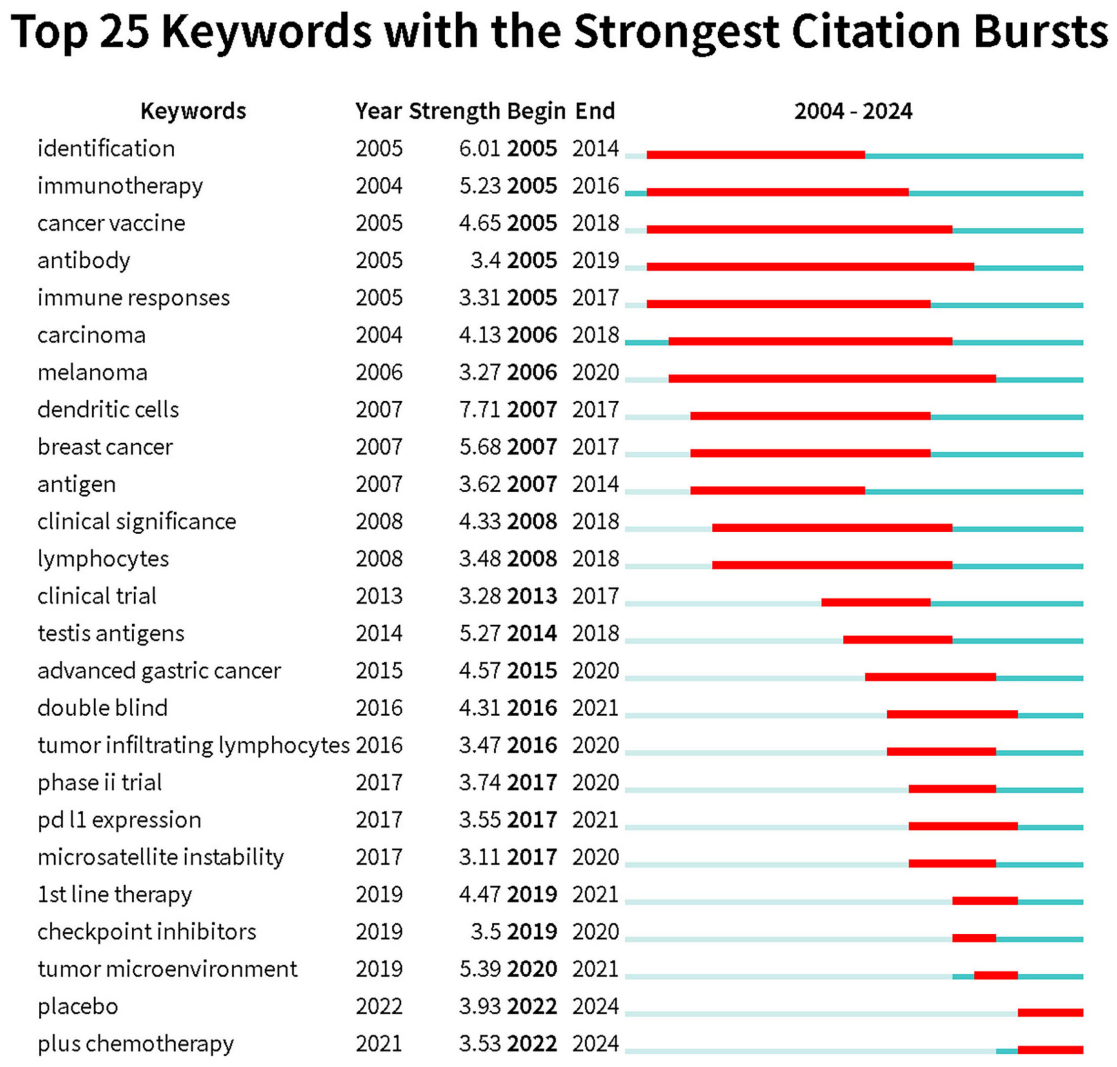
Keywords with the strongest citation bursts related to immunotherapy for EC from 2004 to 2024. The blue line represents the time axis, with the red part indicating the start year, end year, and duration of the burst.

Keyword burst refers to a keyword experiencing a sudden surge in citations within a short period. By analyzing burst keywords, we gain insights into the development trends in esophageal cancer immunotherapy research. We specifically examined the 25 most frequent burst keywords from 2004 to 2024. The term “immunotherapy” was already appearing frequently as early as 2005 and continued to rise until 2016. In recent years, the most frequent burst keywords are “plus chemotherapy,” “placebo,” and “tumor microenvironment” (**Figure 8**, Keyword burst).

### 3.5 Analysis of co-citation references and co-citation reference burst analysis

**Figure 9:**
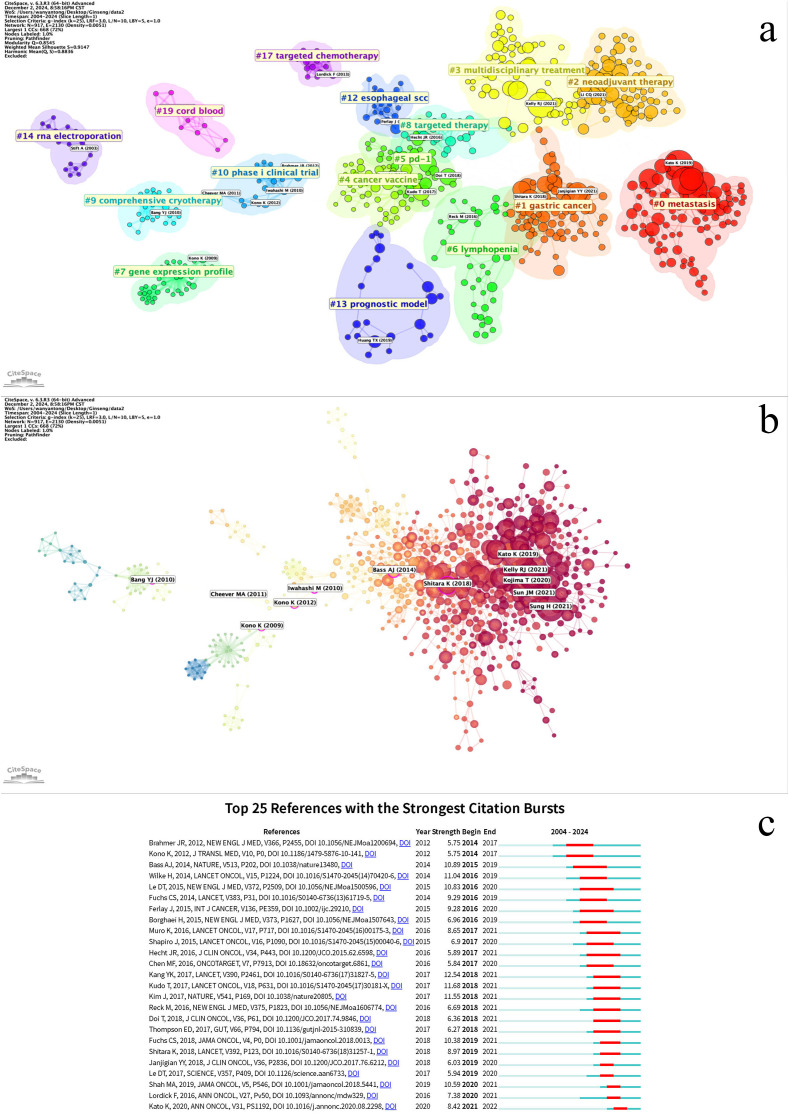
**(a)** Cluster co-cited references based on keywords. **(b)** Literature relationship network diagram, with red nodes representing literature with citation bursts. The size of the nodes represents the citation frequency. **(c)** Co-cited references with the strongest citation bursts related to immunotherapy for EC from 2004 to 2024. The blue line represents the time axis, with the red part indicating the start year, end year, and duration of the burst.

The co-cited references can be grouped into 16 clusters (**Figure 9a**, Clustered cited references visualization diagram). Co-cited references are those that are frequently cited together with other publications, and they are considered foundational to the research in a particular field. The smaller the number, the more keywords are contained within the cluster. Cluster #0, labeled *metastasis*, is most closely related to the study by Kato K et al., *“*Nivolumab versus chemotherapy in patients with advanced oesophageal squamous cell carcinoma refractory or intolerant to previous chemotherapy (ATTRACTION-3): a multicenter, randomized, open-label, phase 3 trial”. Cluster #1, labeled *gastric cancer*, is most related to the work by Janjigian YY et al., “Nivolumab plus chemotherapy versus chemotherapy as first-line treatment for advanced gastric cancer/gastroesophageal junction cancer/oesophageal adenocarcinoma (CheckMate 649): a multicenter, randomized, open-label, phase 3 trial”, highlighting the popularity of nivolumab in the 2019–2021 period within this field. Cluster #2, labeled *neoadjuvant therapy*, is closely related to the study by Li CQ et al., “Preoperative pembrolizumab combined with chemoradiotherapy for oesophageal squamous cell carcinoma (PALACE-1)”.In the CiteSpace analysis, relationships between studies are mapped, and references experiencing explosive citation growth are highlighted (**Figure 9b**, References relationship network diagram). The earliest such explosive year is 2009. The number of burst studies and the frequency of citations reached their peak in 2021. A time burst in annual citation frequency often indicates concentrated attention on literature related to a specific direction within the field, reflecting emerging academic trends, new topics, and potential future research hotspots (24).

The top 25 burst citations began to appear in 2014, with the majority concentrated between 2016 and 2021. The study with the highest burst intensity is Kang YK’s 2017 publication, “Nivolumab in patients with advanced gastric or gastro-oesophageal junction cancer refractory to, or intolerant of, at least two previous chemotherapy regimens (ONO-4538-12, ATTRACTION-2): a randomized, double-blind, placebo-controlled, phase 3 trial” (**Figure 9c**, Co-citation reference burst).Co-citation reference burst can be analyzed across three dimensions:1. Timeline Dimension: This progression can be divided into the Exploratory Phase, focusing on monotherapy safety and fundamental mechanisms (e.g., refs 25, 26); the Surge Phase, representing the maturation of clinical translation for immunotherapy, during which burst keywords accounted for 60% of the literature; and the Maturation Phase, characterized by articles deepening biomarker research and incorporating immunotherapy into standard treatment (e.g., refs 27–29). This evolution can be summarized as transitioning from monotherapy safety exploration to breakthroughs in second-/third-line therapy, culminating in the establishment of first-line combination therapy.2. Cluster Dimension: Key clusters include Clinical Efficacy Validation (concentrated burst: 2016-2019), Biomarker Exploration (longest burst duration: 2016-2021), Combination Therapy Strategies (increasing burst intensity, peaking in 2021), and Molecular Basis of the Disease (spanning the entire period: 2015-2021).3. Biomarker Dimension: The focus shifted from single biomarkers to the overall tumor microenvironment (e.g., refs 28, 30).

## 4 Discussion

### 4.1 Perspective of country

Analysis of publications from the top ten countries/regions in esophageal cancer immunotherapy research (2004-2024) reveals a tripartite global research landscape: China dominates in volume with 370 publications (53% of the total) but lags in efficiency (14.48 citations per publication), suggesting a potential quantity-quality imbalance. The United States balances scale with impact (159 publications, 20.38 citations per publication), while Japan achieves exceptional quality (101 publications) driven by its epidemiological and technical expertise, boasting a field-leading citation rate of 37.03 per publication. European nations demonstrate specialized strengths – Spain’s outlier-like citation rate of 41.77 (13 publications) marks highly impactful niche research, while Germany (19.71), England (26.33), and France (23.13) maintain robust per-publication influence. This suggests that China should prioritize enhancing the quality of clinical trials over quantitative expansion in esophageal cancer immunotherapy research. However, attributing this phenomenon solely to perceived quality deficiencies oversimplifies the issue. Structural characteristics of the scientific ecosystem—notably language barriers and insufficient international collaboration, which significantly constrain research dissemination—along with a predominant focus on locally prevalent ESCC and retrospective study designs collectively shape the current citation patterns. The US-Japan models highlight the value of multidisciplinary collaboration, and Europe’s focused excellence (e.g., Spain’s efficiency) confirms the advantage of specialized niches, though Spain’s extreme citation rate warrants verification for potential single-study bias.

Notably, all top ten publishing countries are economically developed with strong comprehensive national power. It has been reported that mortality-to-incidence ratios in low- and medium-Human Development Index (HDI) countries are nearly double those in high-HDI countries, indicating disparities in prevention, early detection, and availability of optimal treatment services (25). Furthermore, esophageal cancer incidence exhibits strong geographic characteristics, with high-risk areas concentrated in East Asia, Eastern Africa, and Southern Africa. Crucially, Eastern and Southern Africa are absent from the top publishing list, underscoring a structural imbalance in the global allocation of medical research resources. Enhancing collaboration between high-incidence regions and research powerhouses could have a transformative impact on the field of esophageal cancer immunotherapy, potentially addressing fundamental blind spots and translational bottlenecks in current research.

### 4.2 Perspective of institution

Analysis of the top 10 institutions in esophageal cancer immunotherapy research (2004-2024) reveals China’s dominant publication volume with significant quality disparities. Zhengzhou univ leads in output (30 articles), yet its citation per article (15.93) ranks mid-table. Fujian med univ (23 articles, 5.17 citations/article) and Hebei med univ (18 articles, 5.50 citations/article) demonstrate low efficiency metrics amid quantitative expansion. Crucially, Shandong univ achieves a disproportionately high 44.44 citations per article from only 16 publications—exceeding the second-ranked institution by 60% and paralleling Spain’s high-impact model in country-level analyses. natl canc ctr (27.83 citations/article) and univ Texas md Anderson canc ctr (18.95) maintain balanced scale-impact profiles.

Underlying inefficiencies persist: Despite publishing 14 more articles than Shandong univ, Zhengzhou univ’s total citations (478) lag behind Shandong univ’s 711, necessitating optimized research focus. Specialized institutions show polarization—Fudan univ’s critically low citation rate (3.40 from 20 articles) indicates clinical translation deficiencies, while Sichuan univ (14.56) and Chinese acad med sci & Peking union med coll (14.43) sustain moderate efficiency through interdisciplinary collaboration. The presence of univ Texas md Anderson canc ctr (360 citations) confirms international competition, yet China’s 80% institutional representation (8/10) underscores its regional dominance.

### 4.3 Perspective of journal

Based on the analysis of the top 10 journals publishing on immunotherapy for esophageal cancer from 2004 to 2024, the following key patterns emerge: Frontiers-series journals (Frontiers in Oncology and Frontiers in Immunology) dominate publication volume with 83 articles (accounting for nearly half of the total), highlighting the appeal of their open-access model for rapid dissemination of clinical and basic research. However, high-impact journals demonstrate a pronounced “quality-over-quantity” characteristic—exemplified by *Clinical Cancer Research* (CCR, Q1, IF=9.0), which published merely 8 articles yet achieved an exceptional 181.6 citations per article (total citations: 1,453), underscoring top-tier journals’ selectivity for groundbreaking studies. Concurrently, specialized journals deliver outstanding performance: *Esophagus* (Q3, IF=2.2) and *Cancer Science* (Q3, IF=2.0) both exceed 11 citations per article, outperforming certain Q1/Q2 journals (e.g., *Cancers* at 7.26), indicating the advantage of targeted reach in niche domains. Notably, open-access journals exhibit divergent influence (e.g., *Cancers* published 34 articles but with lower citation rates), while the *Journal of Gastrointestinal Oncology* (Q2, IF=4.5), despite its limited sample size (10 articles), attained a remarkable 46 citations per article, signaling its deep penetration in specialized research. Submission strategies should balance journal positioning—prioritize high-barrier Q1 journals like CCR for high-impact innovative findings, consider the Frontiers series for rapid publication of basic or clinical studies, while acknowledging the volatility risks associated with small-sample, high-citation cases.

Based on the analysis of the top 10 most cited journals in esophageal cancer immunotherapy research from 2004 to 2024, the data reveals that top-tier comprehensive journals overwhelmingly dominate: the Journal of Clinical Oncology (J Clin Oncol) holds a commanding lead with 2,715 citations, while the New England Journal of Medicine (NEJM, 1,337 citations), Lancet Oncology (Lancet Oncol, 1,303 citations), and Lancet (Lancet, 858 citations) all exhibit high influence, indicating that major clinical breakthroughs such as phase III trials are predominantly published in top-tier journals. Specialty journals demonstrate distinct characteristics: despite its lower impact factor (IF=12.53), Clinical Cancer Research (Clin Cancer Res) still ranks fourth with 1,066 citations, reflecting its advantage in the field of tumor immunology; the International Journal of Radiation Oncology (Int J Radiat Oncol), as the sole radiation therapy journal on the list (465 citations), indicates the research trend in radio-immunotherapy combinations. Basic research journals show limited influence (e.g., Cancer Research with 745 citations, Nature with 660 citations), proving that the field remains primarily clinically oriented. Notably, a discrepancy exists between impact factor and citation count: the International Journal of Cancer (Int J Cancer, IF=5.7) surpasses higher-IF journals like Nature with 505 citations. For manuscript submission strategy: prioritize J Clin Oncol/NEJM for practice-changing clinical outcomes (e.g., groundbreaking data); select Cancer Research/Nature for innovative mechanistic studies (requiring translational medical evidence); focus on Int J Radiat Oncol for radio-immunotherapy research (supported by biomarker validation); and consider Clin Cancer Res as a “cost-effective” option balancing impact and specialty focus—however, note that total citation counts may be skewed by journal publication volume, and per-article citation analysis is recommended for quality calibration.

### 4.4 Perspective of author

Among the top 10 authors by publication volume, 7 are from Japan, 2 from the United States, and 1 from China, indicating Japan’s leadership in esophageal cancer immunotherapy research, especially in clinical trials (such as the application of anti-PD-1/PD-L1 antibodies). Kato K and Kojima Takashi have made substantial contributions to this field and are recognized as leaders. Additionally, Li Feng from China focuses on the tumor microenvironment and immune evasion mechanisms, providing theoretical support for novel immunotherapies. Researchers from the United States are dedicated to exploring combination therapies and basic immunology. Future research should strengthen international collaboration, leveraging the strengths of various countries to foster multidimensional development.

### 4.5 Perspective of co-cited references and highly cited references

Immunotherapy has emerged as a pivotal domain in cancer treatment, particularly for advanced and treatment-refractory malignancies. Kato K (2019) and Kojima T (2020) independently reported clinical trial outcomes demonstrating the efficacy of anti-PD-1 antibodies (e.g., nivolumab) in patients with advanced esophageal cancer (11, 31), thereby validating the clinical feasibility of immunotherapeutic approaches. Currently available immunotherapies for esophageal carcinoma encompass peptide vaccines, adoptive T-cell therapy, and immune checkpoint inhibitors, all of which have yielded significant clinical outcomes in practice (32, 33). This article provides a detailed exposition on immune checkpoint inhibitors, which primarily include PD-1 inhibitors, PD-L1 inhibitors, and CTLA-4 inhibitors. The PD-1/PD-L1 pathway constitutes a critical immune checkpoint, wherein PD-1 acts as the receptor and PD-L1 as its ligand (34). The interaction between PD-1 and PD-L1 is part of the immune system’s regulatory mechanisms. Under normal physiological conditions, the binding of PD-1 to PD-L1 helps maintain immune system balance, preventing T cells from causing damage to healthy tissues (35, 36). However, in cancer, tumor cells often evade immune surveillance by overexpressing PD-L1 (37). The association between PD-L1 expression and cancer prognosis is not yet fully elucidated; in gastric cancer (GC), esophageal cancer (EC), and hepatocellular carcinoma (HCC), high PD-L1 expression correlates with a poor prognosis (19, 38). Some studies indicate that PD-L status significantly impacts the prognosis of advanced EC with positive lymph node metastasis and distant metastasis (19). PD-L encompasses both PD-L1 and PD-L2, and both are expressed in esophageal carcinoma (39, 40). The PD-1/PD-L1 pathway is complex. PD-L1 can also transmit a positive signal through an unknown receptor distinct from PD-1, leading to T-cell proliferation and the induction of specific cytokines such as interleukin-10 and IFN-γ (19, 41, 42). Concurrently, some research suggests that PD-L2 may represent a superior target for immunotherapy (9), with preferential expression of PD-L2 over PD-L1 observed in esophageal adenocarcinoma (43). However, research concerning PD-L2 remains in development; the mainstream ICIs are still PD-1 and PD-L1 inhibitors, and efficacy has also been observed in patients with PD-L1-negative tumors (44, 45). Although anti-CTLA-4 antibodies exhibit no significant toxicity in antitumor therapy (46), they are associated with more frequent and severe adverse events during treatment compared to nivolumab (47), resulting in relatively less frequent use. Interestingly, one study demonstrated that the combination of an anti-CTLA-4 antibody (ipilimumab) and a PD-1 inhibitor (nivolumab) yields superior therapeutic efficacy compared to nivolumab monotherapy (48). The scope of immune checkpoint inhibitors should also include inhibitors targeting pathways that suppress antigen-presenting cells (APCs). Specifically, CD47 expressed on tumor cells interacts with its receptor on M1-like tumor-associated macrophages (TAMs), thereby impairing their phagocytic activity. Consequently, the development of anti-CD47 antibodies is warranted, and anti-CD47 therapy has been shown to enhance the efficacy of PD-1 and CTLA-4 inhibitors in esophageal squamous cell carcinoma (ESCC) (49, 50). Significant breakthroughs have also occurred in the core mechanisms of immunotherapy, moving from broad populations to precise classification. The team of Xu Ruihua/Wang Feng performed whole-exome sequencing on 486 patients and constructed the EGIC system. EGIC comprises three subtypes: EGIC1, EGIC2, and EGIC3. EGIC1 (characterized by high immunogenicity and low-risk variations): 75% of patients derived durable benefit from “PD-1 antibody + chemotherapy”, with a 3-year overall survival (OS) rate exceeding 40%. EGIC2 (characterized by a single favorable feature): demonstrated moderate benefit. EGIC3 (characterized by low immunogenicity and high-risk variations): requires combined targeted intervention. This classification signifies the initial establishment of a personalized trend for immunotherapy in esophageal cancer.

Despite demonstrating significant clinical efficacy in esophageal cancer, immune checkpoint inhibitors still encounter primary or secondary resistance in the majority of patients (51, 52). Within the conventional PD-1 and PD-L1 axis, PD-L1 expression stands as one of the most biologically rational predictive biomarkers for ICI treatment efficacy (32). However, the clinical implementation of PD-L1 immunohistochemical (IHC) testing is challenging. Furthermore, response rates among PD-L1-positive patients vary considerably, highlighting the need for improved biomarker-driven therapeutic approaches (52). Consequently, it is imperative to identify more suitable biomarkers for selecting patients likely to respond to these agents and to develop combination therapies to overcome resistance (53). Emerging reports suggest that inhibition of TIGIT(T cell immunoreceptor with Ig and ITIM domains) has shown promise in enhancing anti-tumor immunity by complementing the PD-L1/PD-1 axis. Early clinical data indicate that adding anti-TIGIT agents to existing regimens yields superior responses compared to chemotherapy alone or conventional chemoimmunotherapy involving PD-L1 blockade (54). Infiltration of CD69^+^CD8^+^ tissue-resident memory T (TRM) cells is strongly associated with outcomes following neoadjuvant immunochemotherapy (nICT), suggesting TRM could serve as a prognostic biomarker superior to pathological complete response (pCR). Peripheral blood indicators also hold potential as novel biomarkers, such as the ctDNA clearance window period. A growing body of evidence indicates that mRNA splicing-derived neoantigens possess substantial potential as immune targets, potentially significantly improving outcomes for cancer patients. Although significant progress has been made in expanding immunotherapy targets using RNA-sequencing (RNA-seq) technology, considerable work remains needed (55). In esophageal cancer, high PD-L1 expression serves as a significant independent prognostic factor for patients with esophageal squamous cell carcinoma and high HLA(Human Leukocyte Antigen) class I expression (56). In head and neck squamous cell carcinoma (HNSCC), PD-L2 has been reported as an important predictor of progression-free survival for pembrolizumab, independent of PD-L1 status (57). The 2015 report by Shapiro J demonstrated that neoadjuvant chemoradiotherapy followed by surgery yields superior efficacy compared to surgery alone (58). The study by Van Hagen P further elucidated the role of neoadjuvant chemoradiotherapy in the treatment of locally advanced esophageal cancer, providing a theoretical foundation for its combination with immunotherapy (59). Research by Doki Y and Sun JM evaluated the combined effects of immunotherapy with traditional treatment strategies (20, 21), demonstrating its potential in both locally advanced and metastatic esophageal cancer. The converging evidence from these studies paves the way for the standardization of immunotherapy approaches. For patients deemed inoperable, consolidative therapy with camrelizumab following definitive concurrent chemoradiotherapy (dCCRT) achieved a 3-year overall survival (OS) rate of 66.6% and a 3-year progression-free survival (PFS) rate of 62.5% in those with unresectable locally advanced esophageal squamous cell carcinoma. These outcomes are substantially higher than historical data. Future treatment strategies for esophageal cancer will increasingly rely on combination therapies and the identification of novel biomarkers.

Future research directions will focus more intensively on overcoming resistance and translating mechanistic insights into clinical applications. 1. Combination Therapy and Personalized Precision Medicine: Exploration of chemotherapy combined with immunotherapy is progressively increasing, with existing reports demonstrating that the efficacy of combined immune checkpoint inhibitors and chemotherapy is generally superior to chemotherapy alone (60). Concurrently, the combined application of radiotherapy and immunotherapy continues to evolve. Personalized precision medicine is also developing steadily, aiming to achieve better outcomes in enhancing efficacy, reducing side effects, and prolonging survival. 2. Personalized Vaccines and Neoantigen Therapies: MUC1 mRNA vaccine: Achieved an antigen-specific T-cell activation rate of 82% and prolonged progression-free survival (PFS) by 300% in preclinical models. Neoantigen dendritic cell vaccine: Currently undergoing phase II clinical trials in combination with immune checkpoint inhibitors. 3. Real-World Data Driving Value-Based Care: Global collaboration is establishing a molecular subtyping database for resistance and a multicenter registry system for immune-related adverse events (irAEs). These initiatives guide updates to the CSCO(Chinese Society of Clinical Oncology) guidelines and drive the advancement of personalized treatment. The key challenges lie in validating efficacy and conducting cost-effectiveness analyses.

### 4.6 Perspective of keyword

Through keyword statistics, esophageal cancer and immunotherapy emerge as the core themes of this study, with a specific focus on esophageal squamous cell carcinoma as a subtype. The frequent appearance of these keywords reflects the varying responses of different tumor subtypes to immunotherapy, highlighting the need for individualized research for each subtype. Traditional treatment methods for esophageal cancer, such as radiation therapy (RT) and chemotherapy, are no longer sufficient for advanced or chemotherapy-refractory esophageal cancer patients. Consequently, new treatment options such as targeted therapy, immunotherapy, and neoadjuvant therapy are increasingly being explored. The concept of immunotherapy dates back to the late 19th and early 20th centuries. However, significant breakthroughs occurred after 2010, especially with immune checkpoint inhibitors. In 2011, ipilimumab was approved by the FDA(Food and Drug Administration) for the treatment of advanced melanoma, followed by the approval of pembrolizumab and nivolumab (both PD-1 inhibitors) in 2014, ushering in the era of immune checkpoint inhibitors. Currently, research on immune checkpoint inhibitors like PD-1 inhibitors, PD-L1 inhibitors, CTLA-4 inhibitors, and the tumor immune microenvironment has become a hot topic. Future research directions should focus on combination therapies and personalized precision therapy. Currently, explorations of chemotherapy combined with immunotherapy are gradually increasing, and it has been reported that the efficacy of immune checkpoint inhibitors combined with chemotherapy is generally superior to chemotherapy monotherapy (60), while the combined application of radiotherapy and immunotherapy continues to develop, and personalized precision therapy is also gradually advancing.

#### 4.6.1 Keyword burst analysis

Burst keywords can reflect the latest research hotspots in a given academic field. To highlight recent trends, we created a keyword burst analysis for the period of 2004-2024. The keyword with the highest burst intensity is “dendritic cells,” which is unsurprising, as dendritic cells play a crucial role in initiating immune responses. Dendritic cells are antigen-presenting cells, and in immunotherapy, PD-1 inhibitors and PD-L1 inhibitors work by reversing the suppression of dendritic cell function via the PD-1/PD-L1 pathway (61). Similarly, CTLA-4 inhibitors operate in the same manner (62), thereby promoting anti-tumor immune responses. Given their foundational role in immune responses, dendritic cells may become a key focus of future immunotherapy. Immunotherapy, considered a “savior” for advanced esophageal squamous cell carcinoma patients who are refractory to or intolerant of chemotherapy, saw a peak in interest from 2005 to 2016. During this period, keywords like “tumor immune microenvironment,” “immune checkpoint inhibitors,” “PD-L1 expression,” and “microsatellite instability” experienced bursts. The tumor immune microenvironment is a dynamic, multi-layered system, with tumor cells and immune cells playing crucial roles. Immune evasion mechanisms are key factors in tumor development, and further research into the tumor immune microenvironment has provided an important theoretical foundation for the development of immunotherapy (63). Microsatellite instability (MSI) and PD-L1 expression levels are key indicators of the tumor immune microenvironment, with high PD-L1 expression often associated with poor prognosis in esophageal cancer (64). Tumors with high MSI typically exhibit stronger immunogenicity, which may make them more sensitive to immune checkpoint inhibitor therapy, influencing the effectiveness of immunotherapy (2). Immune checkpoint inhibitors have been a major focus in recent years, particularly the advent of pembrolizumab and nivolumab, which have significantly impacted the treatment strategies for advanced esophageal cancer. These developments highlight the growing importance of immunotherapy in the therapeutic landscape of esophageal cancer.

## 5 Conclusion

This study analyzes global academic research on esophageal cancer immunotherapy. The rapid increase in publications in recent years signifies growing researcher interest, establishing it as a key focus in esophageal cancer treatment. China, Japan, and the United States should enhance communication and collaboration to sustain their substantial contributions to the field, while China, in particular, must transition from quantity-driven publication practices towards genuine value creation. Frontiers in Oncology ranks first in publication volume, whereas the Journal of Clinical Oncology (J Clin Oncol) leads in co-citation frequency. Researchers may publish esophageal cancer immunotherapy findings in these journals or consult their content to stay abreast of advancements; for rapid dissemination, Frontiers in Oncology should be prioritized, whereas seminal breakthrough discoveries warrant submission to premier journals. Kato Ken and Kojima Takashi are the most prolific scholars in this field. Researchers focused on esophageal cancer immunotherapy should pay close attention to their work to gain deeper insights into the progress and trends in this area. Overall, despite esophageal cancer being a globally prevalent and high-mortality malignancy, with advanced or recurrent cases having a poor prognosis and a five-year survival rate below 20%, immunotherapy has improved survival rates for advanced or recurrent esophageal cancer. Immunotherapy has gradually transitioned into a more personalized and precise approach, with treatment plans tailored to individual patient needs. Identifying suitable biomarkers is crucial for the precision of immunotherapy. Researchers have proposed four distinct types of tumor immune microenvironment (TIME) based on factors such as tumor-infiltrating lymphocytes and PD-L1 expression, which could help design optimized immunotherapy or combination treatment strategies (51, 65).

Future research directions will place greater emphasis on overcoming therapeutic resistance and translating mechanistic discoveries into clinical applications.1. Combination Therapy and Personalized Precision Medicine: Current exploration of combining chemotherapy with immunotherapy is progressively increasing. Published reports demonstrate that the efficacy of combined immune checkpoint inhibitors and chemotherapy regimens is generally superior to chemotherapy alone (60). Concurrently, the combined application of radiotherapy and immunotherapy continues to evolve. Personalized precision medicine is also advancing steadily, aiming to achieve superior outcomes in enhanced efficacy, reduced adverse effects, and prolonged survival.2. Personalized Vaccines and Neoantigen Therapies, MUC1 mRNA Vaccine: Achieved an antigen-specific T-cell activation rate of 82% and prolonged progression-free survival (PFS) by 300% in preclinical models. Neoantigen Dendritic Cell Vaccine: Currently undergoing Phase II clinical trials in combination with immune checkpoint inhibitors.3. Real-World Data Driving Value-Based Care: Global collaborative efforts are establishing a molecular subtyping database for resistance mechanisms and a multicenter registry system for immune-related adverse events (irAEs). These initiatives guide updates to the CSCO guidelines and drive the advancement of personalized treatment strategies. The primary challenges in this area lie in validating clinical efficacy and conducting rigorous cost-effectiveness analyses.

### 5.1 Ethical statement

This bibliometric analysis utilized exclusively publicly accessible, pre-aggregated data from the following open repositories: Web of Science Core Collection. No individual-level data, patient records, or sensitive identifiers were accessed or processed. The authors declare no conflicts of interest related to data sources or analytical tools.

### 5.2 Limitation

This study has certain limitations. The included literature may not be comprehensive, as it primarily comes from the Web of Science Core Collection database and is limited to English-language publications. Therefore, the results may not fully represent all the research in the field of esophageal cancer immunotherapy. However, given the large sample size of 780 publications, this study remains highly representative and provides valuable insights for academic research in this area.

In conclusion, the research on esophageal cancer immunotherapy shows promising development, and collaboration among countries, institutions, journals, and authors is a key driving force for its future progress. At the same time, addressing the current research hotspots and their limitations will likely further deepen future research directions, promoting innovation and practical applications in this field.

## Data Availability

The original contributions presented in the study are included in the article/supplementary material. Further inquiries can be directed to the corresponding author/s.
